# Market making and the production of nurses for export: a case study of India–UK health worker migration

**DOI:** 10.1136/bmjgh-2023-014096

**Published:** 2024-02-28

**Authors:** Sibille Merz, Benjamin M Hunter, Susan F Murray, Ramila Bisht

**Affiliations:** 1 Department of International Development, King's College London, London, UK; 2 School of Social & Political Sciences, University of Glasgow, Glasgow, UK; 3 School of Global Studies, University of Sussex, Brighton, UK; 4 Centre of Social Medicine and Community Health, Jawaharlal Nehru University, New Delhi, India

**Keywords:** Qualitative study, Public Health, Health policy

## Abstract

**Background:**

High-income countries increasingly look to the international recruitment of health workers to address domestic shortages, especially from low-income and middle-income countries. We adapt conceptual frameworks from migration studies to examine the networked and commercialised nature of the Indian market for nurse migration to the UK.

**Methods:**

We draw on data from 27 expert interviews conducted with migration intermediaries, healthcare providers and policymakers in India and the UK.

**Findings:**

India–UK nurse migration occurs within a complex and evolving market encompassing ways to educate, train and recruit nursing candidates. For-profit actors shape the international orientation of nursing curricula, broker on-the-job training and offer language, exam and specialised clinical training. Rather than merely facilitate travel, these brokers produce both generic, emigratory nurses as well as more customised nurses ready to meet specific shortages in the UK.

**Discussion:**

The dialectic of producing emigratory and customised nurses is similar to that seen in the Post-Fordist manufacturing model characterised by flexible specialisation and a networked structure. As the commodity in this case are people attempting to improve their position in life, these markets require attention from health policy makers. Nurse production regimes based on international market opportunities are liable to change, subjecting nurses to the risk of having trained for a market that can no longer accommodate them. The commercial nature of activities further entrenches existing socioeconomic inequalities in the Indian nurse force. Negative repercussions for the source healthcare system can be anticipated as highly qualified, specialised nurses leave to work in healthcare systems abroad.

**WHAT IS ALREADY KNOWN ON THIS TOPIC**

- High-income countries (HICs) increasingly look to the recruitment of healthcare workers from low-income and middle-income countries (LMICs) to fill domestic labour shortages.
- Commercial brokers often mediate health worker migration in LMICs.

**WHAT THIS STUDY ADDS**

- A complex and evolving industry is involved in the education, training and recruitment of Indian nurses seeking to migrate with services driven by profit orientation and often fluctuating international labour market demands.
- The education and training of health workers encompasses more than clinical skills as it also involves social and cultural competencies; we thus refer to a migrant production process seeking to transform nurses into fully fledged workers.
- Actors in the field increasingly produce nurses for specific employer or labour market requirements in HICs such as the UK, further reorienting nursing education and training away from domestic health needs.

**HOW THIS STUDY MIGHT AFFECT RESEARCH, PRACTICE OR POLICY**

- Market making in health worker migration is a concern for health policy-making as the skills taught to nurses seeking to migrate are governed by market opportunities rather than domestic needs; the commercialisation of services may exacerbate inequalities in the Indian nurse force and negative repercussions for the source healthcare system are likely if a flow of highly qualified nurses leave to work in better-resourced healthcare systems abroad.

## Introduction

The projected global shortfall of health workers is predicted to be 10 million by 2030,^1^ the majority in low-income and middle-income countries (LMICs). Migration plays a central part in this as high-income countries (HICs) increasingly look to the international recruitment of health workers to address their domestic shortages. Since November 2021, for example, the UK has signed agreements with India, Kenya, Malaysia, Nepal, the Philippines and Sri Lanka to facilitate the recruitment of health workers.^2^ Driven by poor working conditions and low pay, an increasing number of health workers in LMICs migrate to wealthier countries in the hope of a better life.^3–5^ The WHO estimates that around 15% of health and care workers are currently working outside their country of birth or the country where they gained their first professional qualification.^6^

India is an important case among the world’s suppliers of nurses; in 2017, around 56 000 India-trained nurses worked in the USA, UK, Canada and Australia, corresponding to roughly 3% of the registered nurse force in India.^7^ In the last 5 years, the annual number of Indian nurses and midwives joining the UK register has increased rapidly; there are now 48 395 Indian nurses registered to practice in the UK.^2^ A growing number of middle-income countries are aiming to replicate this as a strategy for economic development; thus, events taking place in India’s nurse training and migration sector carry global significance.

Some countries actively promote outward migration of domestically trained health workers as an economic strategy. For example, the government of the Philippines has perpetuated a culture of out-migration through the commercialisation of the nursing education sector and a laissez-faire attitude towards increasing internal health workforce imbalances.^8 9^ The framing of these nurses as potential sources of remittances, a driver of economic growth, has inspired a set of formalised policies encouraging migration.^10 11^ The South Indian state of Kerala has similarly promoted the migration of nurses. The latest incarnation of this policy is the government’s provision of free training to tribal nursing professionals to increase their chances of finding overseas employment.^12^ This stands in stark contrast with the cost of health worker migration for LMICs; Saluja *et al*,^13^ for instance, have estimated that LMICs lose US$ 15.86 billion (or £12.92 billion) annually due to excess mortality associated with physician migration with the greatest overall cost incurred by India, Nigeria, Pakistan and South Africa.

Existing policy research in this area has aimed to understand key drivers for, and policy responses to, outward migration of health workers,^3–5^ governance frameworks^14 15^ and issues pertaining to regulation and policy formation.^15 16^ Others have focused on the experiences of the migrant workers themselves, including experiences of discrimination in their host countries.^17–19^ With the increase in commercial activities geared towards health worker education for labour migration,^8 20–22^ social science researchers have also begun to examine the processes and actors involved in this market development. The latter often comprise an array of private and public providers^23^ including nursing schools.^8^

Indeed, Deshingkar^24^ has argued that an understanding of contemporary migration is incomplete without a robust understanding of brokers and their networks in recruiting, training and organising for migration pathways. How do these brokers actively produce migrant workers such that they become ideal migrant workers, ‘packaged’ or ‘positioned’ to match specific employer or labour market requirements in HICs? Existing research has flagged some of the facilitatory roles performed by commercial brokers in mediating nurse migration.^25 26^ For the Indian context, Khadria^20^ (p. 1429) has found that such brokers are being used in both training and recruiting health workers, following a ‘business processing outsourcing’ model to deliver services to clients in HICs. Walton-Roberts, in her analysis of India–Canada nurse migration,^25^ refers to the requirements in Canada for particular clinical knowledge and how these requirements create scope for involvement by intermediaries and other actors as part of wider ‘regimes of skill’.

In this article, we build on these and other scholars in migration studies^24–26^ to present an in-depth case study of the actors, practices and processes producing Indian nurses for both the international labour market and for the specific requirements of the National Health Service (NHS) in the UK. By examining primary data from expert interviews with a range of intermediaries, healthcare providers, policymakers and public health experts in both countries, the article sheds light on the increasingly complex and commercialised nature of the nurse migration industry involved, and the proliferation of different kinds of markets and agents seeking to position themselves in the field. Using the India–UK pathway as a case study, the article argues that migration intermediaries do not simply ‘recruit’ but actively produce a nurse force that is both transnationally deployable and tailored to meet specific labour market requirements in HICs. We suggest that it may be useful to conceptualise such developments as similar to those that occurred in manufacturing’s transition from Fordism to Post-Fordism. The article concludes by pointing to the implications for global health policy–making that its empirical and conceptual insights warrant.

## Methods

This article reports on a case study embedded in a larger research project ("*Analysing the transnational provisioning of services in the social sector: the case of commercialisation of NHS services in China and India*") that investigated trade in health-related services with a focus on UK–India and UK–China engagements. The wider study aimed to analyse the key drivers, actors, markets and supporting social and political infrastructures of these trade relations. As part of this, we investigated transnational labour sourcing as a key area of the increasingly commercialised transnational provisioning of healthcare between India and the UK. The Consolidated criteria for reporting qualitative research (COREQ) checklist was used to report the results of this study (see online supplemental appendix 1).

10.1136/bmjgh-2023-014096.supp1 Supplementary data

### Sampling and recruitment

Findings in this article are primarily drawn from 27 expert interviews conducted between 2021 and 2023 (see table 1). Inclusion criteria for interview were: holding a managerial role within nursing labour sourcing or providing healthcare organisations with specific India/UK responsibilities (recruiters or brokers); being a member of senior management of such an organisation with wide-ranging expertise and knowledge of the sector; working in consultative positions with in-depth knowledge of the nursing labour sourcing sector, including for health policy-making; or working for a nursing advocacy organisation or government with a focus on nursing labour sourcing.

**Table 1:** Sample description

Type of respondent	India	UK	Total
Government representative		3	3
Healthcare consultant	2		2
Healthcare provider	4	4	8
Manager, nursing labour sourcing	9	1	10
Representative, nursing advocacy organisation	3	1	4
Total	**18**	**9**	**27**

Respondents were identified through a structured online search of key institutions and actors in the field of international labour sourcing with a specific focus on India. This included a review of the NHS list of ethical recruiters, which also featured India-based recruitment agencies and UK-based agencies focusing on India. At the time, this list included 23 agencies sourcing nurses from India. Where their contact information was available, we contacted the person responsible for the agency’s India operations (for UK-based agencies) or UK operations (for India-based agencies). Where we did not have this information or a geographical division of responsibilities did not exist, we contacted the institution’s managing director. 9 of the agencies on the NHS list had relocated or ceased to exist, had shifted their geographical focus or did not provide contact details. Of the remaining 14 eligible agencies from the list, 5 did not respond to our invitations and 2 individuals initially agreed to participate in an interview but did not attend; no reason for this was given. We eventually held interviews with 7 representatives of these agencies. 3 additional recruiters were identified through snowball sampling, all of whom took part in interviews; one was headquartered in the UK. The majority of respondents in India were based in two hubs for international nurse migration, Kochi and the Delhi NCR region, and we focused our India fieldwork in these locations.

Respondents in the UK focusing on labour migration, including from India, were drawn from the comprehensive sample of 72 UK-based respondents assembled for the larger research project from which this paper stems. Project researchers identified 32 potential respondents who worked for NHS providers in clerical international roles, of whom 20 were successfully recruited into the larger study sample; 8 respondents included in this article were recruited this way, and additional UK respondents with knowledge of India-based nurse migration were recruited through snowball sampling. Primary materials were supplemented by secondary data obtained through structured online searching to verify specific information such as the current pricing of language exams.

‘Experts’ in qualitative research are those recognised as such in their social setting by virtue of their specific knowledge, their community position or their status.^27^ The experts we spoke with spanned the public and private sectors in India and the UK and included mainly representatives from healthcare providers, nursing advocacy organisations and private agencies. Individuals were contacted via email, phone or in person, informed about the nature and objectives of the research and invited to participate. Once they had agreed to participate, respondents were interviewed at a date and time of their choosing, predominantly in their offices or online; two respondents were met in public spaces.

### Data collection

Interviews were conducted in English by SM and BH and followed the tenets of expert interviewing.^27^ After a personal introduction and background information about the interviewers (research expertise and interests, objectives for the project), questions aimed at probing for information about practices and processes in the recruitment of nurses from India. This included questions about key actors in the field; challenges people working in the sector face and any changes in the policy context that have facilitated or hindered international nurse migration (see online supplemental appendix 2). The topic guide was developed based on findings from interviews as part of the larger research project as well as a structured online search about key developments in the field of India–UK health worker migration between 2019 (the year the 2019 NHS Long-term Plan was launched, laying out the strategy for international health worker recruitment) and 2023, using the search terms ‘nurse/nursing’, ‘health worker’, ‘UK’, ‘India’, ‘migration’, ‘recruitment’ and ‘NHS’ connected by Boolean operators. It was also guided by previous work by BH examining brokers in healthcare.^28 29^ Respondents were informed that all data would be anonymised and that they could withdraw from the study any time before the end of the data collection period. Interviews lasted between 40 and 65 min.

10.1136/bmjgh-2023-014096.supp2 Supplementary data

### Data analysis

Due to the commercially sensitive nature of the interview topics, the interviews were not audio-recorded. Detailed notes were taken during and following the interview and subsequently typed up in MS Word. Open questions and key points were discussed among the larger research team and, when necessary, respondents were asked for clarification through follow-up interviews or via email. Final interview notes were analysed thematically^30^ by SM with the help of the qualitative data management software MAXQDA. After early familiarisation with the data, initial descriptive codes were derived by SM through close and repeated reading of the data and discussion with other members of the team. Coded data were subsequently reviewed and grouped according to larger themes, processes and actors. There were three themes, each comprising between one and three subthemes, reflected in the structure of the findings section. An MS Word version of the coding tree has been added as an online supplemental appendix 3. Rigour was maintained through quality control procedures for interviews such as interviewer training, the joint development of topic guides and co-interviewers, and the discussion of codes and findings among the research team.

10.1136/bmjgh-2023-014096.supp3 Supplementary data

### Ethics

Ethics approval for the research was provided by King's College London and is fully compliant with the ethical principles enshrined in the Declaration of Helsinki.

### Patient and public involvement

While patients were not involved in the design or management of this research, it has been developed in cooperation with an advisory network including public and third sector organisations.

### Data availability statement

Interview notes are available via the UK Data Service.^31^

## Findings

A table summarising the findings is provided in online supplemental appendix 4.

10.1136/bmjgh-2023-014096.supp4 Supplementary data

### A nurse migration industry

In India, the evolving and heterogeneous nurse migration industry is comprised of an eclectic mix of public and private actors. The majority of our respondents facilitating nurse migration worked for commercial agencies but some worked for faith-based organisations, professional associations, trade unions and government departments. All focused on the production of nurses for export. They offer a range of services in the education, training and recruitment of nurses. Multi-speciality brokers offer some combination of the brokering of professional placements, course enrolment (usually by agents referred to as ‘education consultants’ who place Indian students in universities abroad), visa services, exam and interview training, and language training for different national requirements. One agency, for instance, worked closely with an English NHS provider and centre for Objective Structured Clinical Examinations (OSCE—a clinical competency test that is part of the UK Nursing and Midwifery Council’s registration process for nurses and midwives trained abroad) to set up their model. It has also bought real estate to house nursing candidates and establish a residential ‘boot camp style programme’ (TNP267); this agency offers a comprehensive package from recruitment to cultural induction and the initiation of social ties, claiming this has led to a 100% retention rate.

While the major share of the cost associated with international migration is borne by UK employers, fees for any additional training or non-essential services, for example supporting the visa application, are borne by the nurses themselves. Most agents we spoke with had somewhat opaque pricing schemes; one suggested (TNP262) that just the additional support for a visa application can cost between INR 7000 and 10 000, or £70–100, roughly a third of an average nurse’s monthly salary. Not least, nurses usually have to pay back at least a share of their relocation cost if they break their stipulated employment contracts within the first 1–3 years; while practices vary between Trusts, this can be between 75% of the relocation costs during the first year and 25% during the third year of their contract (TNP262). From a Trust perspective, this may well be justified given the large investment needed to recruit internationally (respondents estimate the average cost of recruiting an international nurse at between £10 000 and £12 000, for example TNP049); however, for nurses, it means that training for migration and migrating itself carries significant financial risk.

The Indian Ministry of Skill Development and Entrepreneurship has been an active player in the export of nurses too, pledging to supply 300 000 healthcare workers, including nurses, to countries such as the UK, Germany and Australia by 2022.^32^ In addition, many respondents reported that Indian state governments were seeking to boost outward nurse migration, not only for the economic benefit of increasing remittances but also to counter growing youth unemployment or address gender inequalities (TNP267; TNP262). Some state governments have set up their own recruitment wings to exert a degree of control over outward migration. For example, the Kerala government oversees two agencies, the Overseas Development and Employment Promotion Consultants, established in 1977 under the Ministry of Labour, and the Department of Non-Resident Keralite’s Affairs, established in 1996 to address the grievances of Keralites living abroad.^33^ Others, however, seek the support of experienced market-based recruiters with the networks and capital to promote migration in specific areas of employment. For instance, one agency we spoke with had been approached by the state government of Uttar Pradesh, said to be keen on developing a nurse migration programme as part of the state government’s *Nari Shakti* programme to empower young girls and women through education (TNP262).

### The large-scale production of nurses for export

The production of Indian nurses for export involves mechanisms to educate, train and recruit nursing candidates; to facilitate their sponsorship and visa applications; to organise their travel; as well as to provide pastoral care. This specifically involves the international orientation of curricula by nursing colleges, offering on-the-job training by predominantly private providers, as well as language and exam training.

#### Role of nursing colleges in shaping a workforce for export

Nursing colleges play an active role in shaping a workforce for export. Interviews in our study indicated three routes through which this occurs: the international orientation of nursing colleges; collaborations between recruitment agencies and nursing colleges; and, consequently, recruitment agencies’ practice of approaching nursing candidates before they have passed professional licensing exams.

Private nursing colleges have developed explicitly international curricula and orientations. One such example is a newly opened, private college for nursing and allied health professions in Mysore, Karnataka, founded by a recruitment agency. An agent working with the college (TNP267) told us how it employs international faculty (50% of the faculty are from the UK or USA) and uses an ‘international curriculum’ or ‘international pathway’ geared towards the US and UK labour markets but also includes more general guidance on how to secure employment abroad. Offers include instruction for the International English Language Test System (IELTS) or the Occupational English Test (OET) as well as an NHS Orientation module and training for the OSCE. While the college is not exclusively focused on educating nurses for export, the majority of students choose this international pathway. Another UK-based agent (TNP062) suggested the potential for developing nursing colleges in sending countries such as Kenya, dedicated to meeting the training requirements for one specific country. Though these examples are from the UK context, the phenomenon itself is not restricted to India–UK migration routes: agents in our sample have told us about their plans to extend international orientation to include, for instance, elements of Australian and German clinical conventions and language skills (TNP257; TNP246; TNP269; TNP267) or Japanese language skills (TNP269).

Nursing colleges also enter into collaborations with commercial agencies wherein the latter advertise migratory services to nursing candidates, seeking to encourage aspirations for migration. Where we encountered them, these collaborations were informal, but an India-based agent (TNP265) noted that colleges collaborate willingly with agencies as it gives them a competitive edge, allowing them to market themselves as providing international opportunities for nurses. Agents we spoke with held seminars with nursing students in order to inform them about the opportunities awaiting them abroad and to identify potential recruits (TNP265; TNP267). Collaborations between agencies and nursing colleges are heavily commercialised and usually work on a commission basis; if a nursing student signs up to the recruiters’ agency and secures a placement abroad, the nursing college from which they graduated receives a share of the agency’s commission (TNP265; TNP062).

Such is the penetration of agencies into the college system that many enlist nursing candidates for future migration while they are still at college or studying for their language exam, creating what one agent (TNP257) described as a ‘pipeline’ of recruits. With the security of one lucrative job offer abroad in hand, nurses rarely applied for other jobs abroad. English NHS Trusts are reported to have followed this anticipatory recruitment model for some time, and have the advantage of colonial language links (TNP267). However, respondents told us this was also increasingly popular for destinations where English is not the main working language. For example, Germany has previously struggled to find candidates due to their lack of German language skills. Recognising the opportunity to move into this market, actors in the Indian migration industry are now lobbying for German lessons to be integrated as electives into some Indian nursing curricula (TNP257). A downside is, however, that many candidates end up not passing their language tests, reducing the effectiveness of this model (TNP062).

#### Language schools and exam boards expand their market

Other key players in shaping a nurse force for export are (English) language schools and exam boards. A key requirement for Indian nurses seeking to move abroad is the acquisition of advanced language skills, predominantly English. For example, nurses seeking to work in the UK need to demonstrate a working level of English by a minimum overall level 7.0 on the IELTS. Alternatively, they can choose to sit the OET where a minimum Level B in speaking, listening and reading and Level C+ in writing is required.^34^ Language schools offering coaching have been mushrooming across India. They are prominent in urban hubs for nurse migration such as Kochi and New Delhi, as our fieldwork has shown, where a steady supply of aspirants can be enrolled.

Recruitment agencies look to collaborate with these language schools, for example, making use of a centre’s database of students to contact potential recruits (TNP062; TNP255). One agent explained that some language schools make such data available to agents for a fee; others work on the basis of results—the language school receives a ‘referral’ fee from the agent for each eventual recruit (TNP062). There is also reputational benefit to be had by schools, which can cite to the high proportion of their students who go on to work internationally which is ‘good for marketing’ (TNP062). One respondent (TNP255) described these language schools as 'feeding' centres for recruiters. While no exact figures are available, the same agent estimated that around 70%–80% of the nurses who initially joined their organisation for language training eventually also signed up to their recruitment services.

Further opportunities for revenue generation have been created through the outsourcing of language examination. While the IELTS used to be offered by a UK public body, the British Council, in India it is currently offered by IELTS Australia Pty Ltd, a wholly owned subsidiary of private education provider IDP Education Ltd with centres in 75 cities in India. At the time of writing the fee per test is INR 16 250 (around £160). OET is offered in partnership with various educational institutions, usually private, at multiple sites across India for approximately INR 30 000 (£300).^35 36^ By way of comparison, a Delhi-based recruiter told us that nurses in a government hospital earn around INR 40 000 to 45 000 (between £400 and £450) monthly and only around INR 22 000 (£220) monthly in an average, 200-bed private hospital. Large, internationally accredited hospitals such as Apollo were ‘somewhere in the middle’ and usually paid around INR 30 000 (£300) monthly (TNP257). Fees for the OET thus surpass the average monthly salary of a nurse working in the private sector.

#### Private hospitals provide the experience necessary for migration

Private hospitals provide the experience necessary for migration. A minimum of 1 year of clinical work experience is usually required for nurses seeking employment in the English NHS. As such, Indian (private) hospitals play an important role in the export of nurses since, after graduation, it is here that nurses can gain the clinical experience needed to obtain a job internationally. This allows those hospitals to fill staffing gaps with newly qualified and therefore relatively cheap nurse labour. A Kochi-based agent (TNP247) we spoke with was approached by a private hospital chain to establish a scheme whereby nurses would work in their facility for around 1 year and in turn obtain an ‘experience certificate’ in order to be able to move abroad. In their description, such placements resemble internships rather than full-time and remunerated positions. Another respondent, a Delhi-based nurse activist and public health consultant (TNP213), noted that while everyone had ‘their eyes on the big birds like Apollo’, it was usually the smaller private hospitals that were collaborating with recruitment agencies. In their experience, recruiters and hospitals also shared the recruitment fee paid by the overseas employer in the case of NHS Trusts between £1000 and £2000 depending on the number of intermediaries involved (TNP213).

The downside of this situation for private hospital employers is low staff retention. Recruitment agents’ relationships with private hospitals in India can therefore be fraught: one agent (TNP251) jokingly said that hospital managers would ‘kill’ any agent who tried to recruit among the hospital’s staff. Another respondent, a Delhi-based healthcare provider who had just lost his head nurse to a post in Canada, noted that the ‘nurse drain’ was a huge problem since ‘we have a crisis here’ (TNP268). Hospital managers have also reportedly refused to issue reference letters to nurses in an attempt to prevent them from leaving abroad (TNP251). This has created a form of dependency for junior nurses working in the private sector and seeking to apply internationally. A reverse development, however, is that some private hospitals have reportedly increased nurses’ salaries in order to retain them (TNP262)

### Niche production of customised nurses

While the actors and processes described above claim to train and prepare nurses for a broadly framed global career, in practice, training is often geared towards specific countries and the creation of customised workers ready to meet specific shortages or working environments.

#### Specialised and simulation-based training

Bespoke offers include educational and practical, also simulation-based, training. For instance, one agency we spoke with had developed a special foundation programme for mental health to meet demand from UK organisations for mental health nurses (TNP267). The creation of a dedicated training programme was necessary because of the absence of specialised mental health nursing training in the Indian curriculum, and the social stigma around mental health in the country. As the agents struggled to identify certified mental health nurses for the UK labour market due to this absence, they developed the idea for the programme in order to smooth the process and fulfil the NHS requirements. At the time of the interview, they were about to place 120 mental health nurses across different providers in the UK, and had created specific mental health training and practical examination modules (ibid).

Training and simulation equipment is also procured on the basis that it adequately mimics equipment used in the target country. One agent (TNP267) had developed a preceptorship programme for clinical care nursing in collaboration with a London-based hospital Trust, with 50% of the programme conducted in India in order to save on training costs. For this, the agent had purchased the same model ventilators used in the Trust’s intensive care unit, enabling nurses to be trained specifically for this clinical infrastructure. This way, the candidates develop highly specialised skills and expertise according to the requirements of UK healthcare providers. Other large recruitment and training organisations such as a large, Chandigarh-based agency have also been reported to be using simulation tactics for specialised training.

#### Soft skill training for country-specific clinical cultures

The migration industry also offers a range of trainings about different clinical cultures in order to shape nurses’ practices and subjectivities according to specific national standards and expectations. Respondents explained that such training is important because of key challenges related to differing clinical cultures between countries; specific soft skills are crucial to caring for patients (TNP269). UK-based respondents reported these to include differing perceptions for example around patient data confidentiality and consent taking (TNP264). They described Indian nurses as often being rather deferential or from a ‘culture of pleasing’ (TNP062), making them very reluctant to raise concerns. This, respondents argued, could provide a fertile ground for errors, ultimately compromising patient safety. Many Indian health workers thus ‘run into trouble’, as one Indian recruiter (TNP264) stressed, during their first 6 months in the UK. As a response to this, a range of programmes have been developed that focus on cultural dimensions of living and working in the UK and specifically in the NHS. Such programmes are not just aimed at helping nurses settling into their new communities, but also at instilling the values and practices of the NHS in them.

These programmes range from month-long induction packages to the plans for a ‘finishing school’ for nurses and allied health professionals in order to learn ‘how to behave in a culturally appropriate way’ in the receiving country (TNP264). The focus here, then, is not on nurses’ clinical skills but on the embodiment of the values of nursing in the target country, moulding nurses according to a specific country’s or institution’s values and practices. Rather than focusing on verifiable skills or competencies, such training targets subjective traits and personal characteristics such as being less deferential. In brief, nurses need not only master clinical and language skills but also be well-versed in their host country’s social and cultural competencies. One agent (TNP269) suggested that their company charges around INR 20 000–30 000 (£200–300) for such soft skill and clinical training though this cost is usually covered by the overseas employer.

## Discussion

The recruitment of Indian nurses for the UK is made possible by an evolving array of markets and services, each targeting either a specific or a variety of specialised services. Connected through a networked structure of agents and other, predominantly private actors, these services are driven by profit orientation and often fluctuating international labour market demands. Recruitment thereby involves more than merely the facilitation of migration but can be described as a migrant production process, lasting from just a few weeks to several years, seeking to transform nurses or nursing candidates into emigratory workers. While the mutual recognition of qualifications and degrees provides the policy framework for this process,^37^ a transnationally competitive nurse not only requires verifiable clinical and language skills, but must also embrace the cultural values and practices of their host country and institution. In this sense, the process involves the production of nursing subjectivities, that is, particular positions of identity and agency according to dominant forms of knowledge and practice.^38 39^ Here our work corroborates findings from earlier work in migration studies in Indonesia and the Philippines that the shaping of the ideal migrant worker can include an extensive set of attributes such as the gender of workers,^40^ and even extends to subjective attributes such as being docile or hardworking.^41^

This production of nurses involves both the large-scale production of emigratory nurses and the niche production according to nationally specific criteria and standards. Indeed, this seems to resemble Post-Fordist production in manufacturing: a model characterised by flexible specialisation, decentralised management and a networked structure for manufacturing accompanied by a global orientation.^42 43^ Garment manufacturers such as Benetton, for example, derive their success from adaptation to market trends and the networked production through a range of specialist subcontractors concentrating on niche production.^44^ While their clothes are still mass-produced, smaller batches are tailored to specific market demands such that nice production sits amidst a wider, mass-production environment (ibid.). There has been some discussion of Post-Fordism in the context of healthcare in HICs and changes in the nature of the welfare state, specifically the introduction of market-oriented reforms in healthcare provisioning in Israel and the UK.^45 46^ Here we suggest its applicability to increasingly flexible and globalised healthcare labour processes, as the demand for workers is no longer seen as only a mass market but a fragmented one that is best served by specialised services and ultimately workers. The migration process resembles an assembly line that produces both generic emigratory and customised workers in line with a Post-Fordist production model. These types of workers are not always clearly distinguishable; there is significant overlap in that nurses trained for the UK context may become more competitive for other contexts, too, testament to the increasing step-migration of health workers.^47^

The production of nurses is not a process of one-sided commodification, as it furnishes (some) nurses with a new kind of agency, with benefits for those who migrate and who often have to pay back educational loans. However, there are several potentially negative repercussions for the nurses. First, the competencies taught to nurses are governed by international market opportunities and the profits that can be made through these. This may suit nurses who leave India, but the flexibilities intrinsic to Post-Fordist production systems might mean that nurses could find themselves trained to work in a market that no longer wants them. Further, these flexibilities afford substantial leverage to buyers in HICs to make new demands at short notice, or even shift production to other settings. This also leaves suppliers in LMICs in a state of dependency and raises the spectre that they specialise in markets or services no longer needed.

Second, given the commercial nature of these markets, the various services we have described are offered at a significant financial cost. The UK adheres to the WHO Global Code of Practice for International Recruitment^6^ such that the receiving healthcare institution is expected to cover the largest share of the cost of training and travel. However, fees for additional training as well as services related to the visa application process are borne by the nurses themselves. As noted in the findings, costs to be borne by the nurses can include items like INR 16 250 for IELTS and INR 7000–10 000 for visa application fees. For context, nurses in an average private hospital in India earn in the region of INR 22 000 monthly (TNP257). Even those costs covered by future employers such as flights may often have to be initially advanced by nurses, who therefore incur large debts (on debt-financed migration of nurses, see Walton Roberts and Rajan^48^), while employers in the UK include contractual clauses to repay a share of relocation costs in the event of leaving employment within 1–3 years. This means that for nurses, international migration also poses the risk of indebtedness. Moreover, international migration can increase the risk of exploitation within India given the requirement of hands-on clinical experience which incentivises working for very low or no remuneration in order to acquire this experience. This form of exploitation has existed in the healthcare sector prior to the large-scale migration of nurses (Walton-Roberts *et al*,^48^ p. 516) but may even increase with the surge of international labour market demands. In our case study, healthcare providers contribute to the precarisation of nursing labour by creating forms of employment that are more akin to internships or placements, unpaid but necessary to find employment abroad.

Overall, these developments may further entrench existing inequalities among the Indian nurse force: those without language skills, cultural capital or the financial means to pay for additional training or to recoup the cost of relocation are left behind with little opportunity for specialised exposure. This may not only add to already existing inequalities according to class and caste (also see Gill^49^), but also have negative repercussions for the source healthcare system as highly qualified nurses increasingly emigrate to work elsewhere and potential training resources are redirected. Efforts exist at both the state and the federal level to counter the shortage of nurses in India but regional disparities remain. For instance, a current central government scheme aims to establish 157 nursing colleges providing full degrees as well as short-term training opportunities but this scheme has not been utilised equally by all states.^50^ Concern has been raised over the poor uptake of the scheme, especially by states with a chronic shortage of nurses such as Uttar Pradesh (ibid). Several state-level initiatives aim to address gaps and the uneven rural-urban divide in the number of nurses, and human resources for health more broadly; however, these have often failed to be properly monitored and a long-term strategy to increase training opportunities has so far been missing. How the trend to produce both emigratory and customised nurses shapes the development of human resources for health planning and recruitment within India is an important area for future research.

### Strengths and limitations

A key strength of this study is its qualitative approach which has allowed us to obtain data from a relatively large number of representatives from a sector of agents usually difficult to reach given the potential commercial sensitivity of the questions discussed. Similar studies, for example, on pharmaceutical procurement, manufacture and pricing,^51^ have worked with significantly smaller sample sizes. By using fieldnotes rather than recordings, we gained access to a group of companies that are typically difficult to research due to privacy concerns; however, this has also meant we have relied on detailed fieldnotes rather than transcripts in our analysis. For the same reason, findings are not illustrated by longer direct quotations. Our study is also, to our knowledge, the first to have specifically examined India–UK dynamics in international nurse migration. Geographically, the study focused on specific hubs in India to enable local networking and access, and to explore their relationship with the UK. Models and practices identified in this geographical setting might be replicated elsewhere, but this relatively narrow geographical focus may constitute a limitation. Finally, while highlighting their importance, this study has not been able to systematically and quantitatively assess the costs of training, who bears these, and the knock-on effects. There is a need for future research on this aspect.

## Conclusion

We have used the case study of India–UK health worker migration to illuminate the making of new markets in the education, training and recruitment of Indian nurses for international employment. These commercialised processes not only facilitate migration but are more comprehensive in that they aim to furnish nurses with a range of skills and competencies beyond their clinical expertise; they thus actively produce specific nurses for export. The model is not dissimilar to that used in other manufacturing industries. While some actors seek to produce generic emigratory workers, others focus on the niche production of highly customised nurses for nationally specific labour market demands. The skills and competencies taught to nurses are selected based on current international market opportunities which may benefit nurses who seek to leave India, but the often ephemeral and flexible production regimes mean that the skills and competencies are liable to change at short notice. Given the commercial nature of these markets, the training nurses must adopt to secure overseas employments is offered at a significant cost, possibly further entrenching existing social inequalities in the Indian nurse force. This may ultimately also have negative percussions for the source healthcare system as highly qualified, specialised nurses seek to work in better-resourced healthcare systems abroad.

## Data Availability

Data are available in a public, open access repository. Interview notes are available via the UK Data Service.
