# Supplementary material for: Market making and the production of nurses for export: a case study of India–UK health worker migration
Source: BMJ Glob Health. 2024 Feb 28;9(2):e014096. doi: 10.1136/bmjgh-2023-014096 (PMC10910680; doi:10.1136/bmjgh-2023-014096)
Supplement: Supplementary data [file bmjgh-2023-014096supp001.pdf]

Supplementary Material

Consolidated criteria for reporting qualitative studies (COREQ): 32-item checklist

Developed from: Tong A, Sainsbury P, Craig J. Consolidated criteria for reporting qualitative research (COREQ): a 32-item checklist for interviews and focus groups. International Journal for Quality in Health Care. 2007. Volume 19, Number 6: pp. 349 – 357

No	Item and guide question(s)	Guide questions/description
**Domain 1: Research team and reflexivity**		
Personal Characteristics		
1.	Interviewer/facilitator (w*hich author/s conducted the interview or focus group?)*	Interviews were conducted in English by SM and BH following the tenets of expert interviewing
2	Credentials *(what were the researcher's credentials? E.g. PhD, MD)*	All researchers have PhDs in a social science discipline
3.	Occupation (w*hat was their occupation at the time of the study?)*	SM was a postdoctoral researcher on the project with King’s College London; BH was Co-Investigator, project manager and lecturer at the University of Sussex; SFM was Principal Investigator and Professor at King’s College London; RB was Co-Investigator and Professor at Jawaharlal Nehru University.
4.	Gender (*was the researcher male or female?)*	SM: female; BH: male; SFM: female; RB: female
5.	Experience and training (*what experience or training did the researcher have?)*	SM has obtained a PhD in sociology with substantive training and expertise in qualitative methods; BH has a PhD in International Development with a focus on healthcare provisioning in India using qualitative methods; SFM was trained as a midwive and has obtained a PhD in Sociology; she has extensive expertise in researching health-related fields in India and Latin America. RB has a PhD in social sciences in health and has long standing expertise in conducting field research in the public health sector in India; she uses predominantly qualitative methods in her work.
Relationship with participants		
6.	Relationship established (*was a relationship established prior to study commencement?)*	Three respondents had been known to the project team from previous research; all other respondents were contacted for this study.
7.	Participant knowledge of the interviewer (*what did the participants know about the researcher? e.g. personal goals, reasons for doing the research)*	At the beginning of each interview, interviewers gave a brief introduction about themselves, their expertise and their motivations for conducting the project.

8.	Interviewer characteristics (*what characteristics were reported about the interviewer/facilitator? e.g. Bias, assumptions, reasons and interests in the research topic)*	Research expertise and interests, objectives for the project
**Domain 2: Study design**		
Theoretical framework		
9.	Methodological orientation and theory (*what methodological orientation was stated to underpin the study? e.g. grounded theory, discourse analysis, ethnography, phenomenology, content analysis)*	The project uses a theoretical approach on the production of nurses adapted from migration studies; its methodological orientation, an inductive thematic approach following Braun and Clarke, 2021, is outlined in the Methods section.
Participant selection		
10.	Sampling (*how were participants selected? e.g. purposive, convenience, consecutive, snowball)*	Sampling is described in the Methods section.
11.	Method of approach (*how were participants approached? e.g. face-to-face, telephone, mail, email)*	Method of approach is detailed in the Methods section: "Individuals were contacted via email, phone or in person, informed about the nature and objectives of the research, and invited to participate. Once they had agreed to participate, respondents were interviewed at a date and time of their choosing"
12.	Sample size (*how many participants were in the study?)*	27 individuals participated in this part of the study as outlined in the Methods section and in Table 1
13.	Non-participation (*how many people refused to participate or dropped out? Reasons?)*	Out of 23 agencies sourcing nurses from India, nine had relocated or ceased to exist, had shifted their geographical focus or did not provide contact details. Of the remaining 14 eligible agencies from the list, five did not respond to our invitations and two individuals initially agreed to participate in an interview but did not attend; no reason for this were given. Out of the comprehensive sample of 72 UK-based respondents assembled for the larger research project, researchers identified 32 potential respondents who worked for NHS providers in clerical international roles, of whom 20 were successfully recruited into the larger study sample; eight respondents included in this article were recruited this way.
Setting		
14.	Setting of data collection (*where was the data collected? e.g. home, clinic, workplace)*	Data was predominantly collected in respondents' offices or online; two participants were met in public spaces.

15.	Presence of non-participants (*was anyone else present besides the participants and researchers?)*	In one interview, the respondent's wife and co-founder of the company briefly joined the conversation.
16.	Description of sample (*what are the important characteristics of the sample? e.g. demographic data, date)*	A sample description has been provided in Table 1.
Data collection		
17.	Interview guide (*were questions, prompts, guides provided by the authors? Was it pilot tested?)*	A topic guide was used for the interview (see Appendix 2).
18.	Repeat interviews (*were repeat interviews carried out? If yes, how many?)*	No repeat interviews were carried out.
19.	Audio/visual recording (*did the research use audio or visual recording to collect the data?)*	The interviews were not recorded but fieldnotes were taken and typed up by the interviewers. The decision not to record the interviews was made by the project team on the basis of extensive past experience researching private and commercial models of healthcare in multiple settings.
20.	Field notes (*were field notes made during and/or after the interview or focus group?)*	Fieldnotes were taken, typed up and synthesised after the interview.
21.	Duration (*what was the duration of the interviews or focus group?)*	Interviews lasted between 40 and 65 minutes.
22.	Data saturation (*was data saturation discussed?)*	Data saturation was discussed; while additional data from other sites and settings key to Indian health workers' outward migration may have yielded additional insight, this was not feasible due to the limited resources of the project.
23.	Transcripts returned (*were transcripts returned to participants for comment and/or correction?)*	While transcripts were not produced for the study, fieldnotes were sent to participants when they expressed a wish to do so.
**Domain 3: analysis and findings**		
Data analysis		
24.	Numbers of data coders (*how many data coders coded the data?)*	One author coded this subsample of the entire dataset; codes were discussed with the entire team.
25.	Description of the coding tree (*did authors provide a description of the coding tree?)*	A description of the coding tree has been added in Appendix 3.
26.	Derivation of themes (*were themes identified in advance or derived from the data?)*	Themes were identified inductively as outlined in more detail in the Data Analysis section.
27.	Software (*what software, if applicable, was used to manage the data?)*	The qualitative data management software MAXQDA was used.
28.	Participant checking (*did participants provide feedback on the findings?)*	Participants have not provided feedback on the findings.
Reporting		
29.	Quotations presented (*were participant quotations presented to illustrate the themes / findings? Was each quotation identified? e.g. participant number)*	As interviews were not recorded, there are few (long) verbatim quotations but these have been added where possible. Each statement or brief quotation has been identified by a participant number.

30.	Data and findings consistent (*was there consistency between the data presented and the findings?)*	Yes.
31.	Clarity of major themes (*were major themes clearly presented in the findings?)*	Yes. Three major themes have been reported in this study.
32.	Clarity of minor themes (*were minor themes clearly presented in the findings?)*	Yes. Six minor themes were reported in this study.
