# Supplementary material for: Market making and the production of nurses for export: a case study of India–UK health worker migration
Source: BMJ Glob Health. 2024 Feb 28;9(2):e014096. doi: 10.1136/bmjgh-2023-014096 (PMC10910680; doi:10.1136/bmjgh-2023-014096)
Supplement: Supplementary data [file bmjgh-2023-014096supp002.pdf]

Topic guide for interviews on labour sourcing, education and migration of health workers

1. **Introductions and verbal consent process**

2. **To start, could you please just say a little about your organisation and your role within it?**

3. **I am particularly interested in the details of cross-border labour sourcing, education and migration of health workers. Can you tell me more about your activities in this area and how this works in practice?**

   [Possible follow-up questions, depending on organisation, role and collaborations: How do you build relationships with healthcare providers? How do you select partners? How do you advertise? How do you recruit health workers? What services do you provide? How much do you charge for your services and to whom? What other organisations are involved in these processes? What seems to work well? Have any problems arisen? What were the effects of COVID-19 on these activities?]

4. **[Depending on location of respondent] Can you tell me a bit more about your work with organisations in India / the UK specifically? How did that arise?**

   [Possible follow-up questions: who do your work with in the UK – other agencies or directly with hospitals/healthcare providers? Are any other UK organisations involved as partners? How do you find working with organisations in the UK compared to other countries?]

5. **Why do health workers want to work in the UK? Are other destinations preferred and has that changed over time?**

   [Possible follow-up questions: do you find that there is any difference between what people expect when going to the UK, and how it turns out in reality? Are there any problems your clients face when trying to work in the UK specifically? Has COVID changed how people think about the UK/NHS at all?]

6. **I have seen a few UK-India programmes which describe themselves as 'earn, learn and return'. How are these kinds of programmes seen within the sector?**

   [Possible follow-up questions: is there any drawback to this kind of model? Should this kind of model be expanded more widely?]

7. **Finally, are there any specific policy or government changes in India or the UK that have helped (or hindered) your work?**

   [Possible follow-up questions: has Brexit affected your work at all?]

Thanks, opportunity for questions and suggestions for further respondents.
