# Supplementary figures and images for: Market making and the production of nurses for export: a case study of India–UK health worker migration
Source: BMJ Glob Health. 2024 Feb 28;9(2):e014096. doi: 10.1136/bmjgh-2023-014096 (PMC10910680; doi:10.1136/bmjgh-2023-014096)

Appendix 3: coding tree

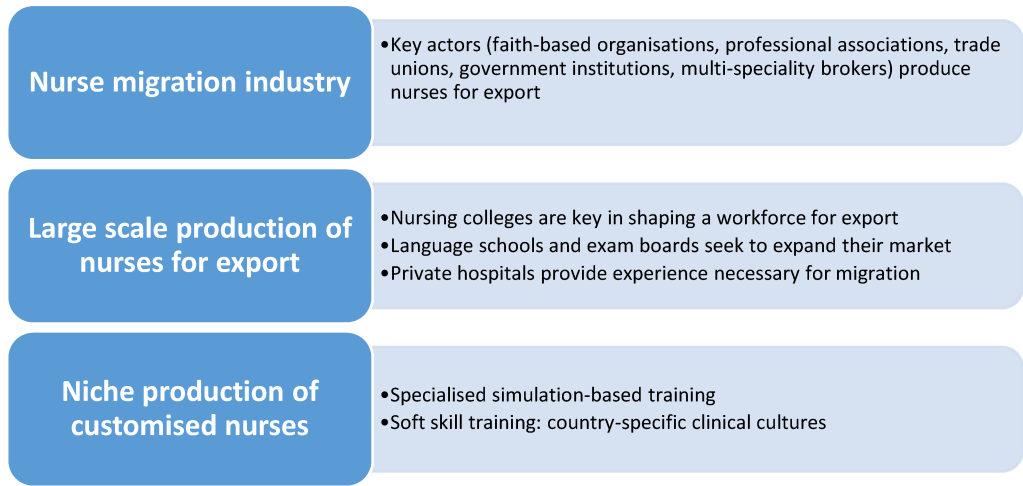

Supplement: Supplementary data [file bmjgh-2023-014096supp003.pdf]
