# Supplementary material for: Market making and the production of nurses for export: a case study of India–UK health worker migration
Source: BMJ Glob Health. 2024 Feb 28;9(2):e014096. doi: 10.1136/bmjgh-2023-014096 (PMC10910680; doi:10.1136/bmjgh-2023-014096)
Supplement: Supplementary data [file bmjgh-2023-014096supp004.pdf]

Appendix 4: Summary Table

Theme	Finding
Constitution of a nurse migration industry	A complex industry is involved in the education, training and recruitment of Indian nurses seeking to migrate, with services driven by profit-orientation and often fluctuating international labour market demands. The education and training of health workers encompasses more than clinical skills but also involves social and cultural competencies; it is therefore a migrant production process seeking to transform nurses into fully-fledged workers.
Large scale production of nurses for export	Nurses are produced for export through various mechanisms to educate, train and recruit nursing candidates, facilitate their sponsorship and visa applications, organise their travel as well as provide pastoral care. More specifically, this production process involves the international orientation of curricula by nursing colleges, language and exam training, and on-the-job training by predominantly private providers.
Niche production of customised nurses	Actors in the field increasingly produce nurses for specific employer or labour market requirements in high-income countries such as the UK. Training offered includes specialised and simulation-based training as well as soft skills training required by UK providers. This tailored approach specific risks for nurses and suppliers given the volatility of post-Fordist production regimes.
